# Isolation and identification of microflora from the midgut and salivary glands of *Anopheles* species in malaria endemic areas of Ethiopia

**DOI:** 10.1186/s12866-019-1456-0

**Published:** 2019-04-29

**Authors:** Abib Berhanu, Adugna Abera, Desalegn Nega, Sindew Mekasha, Surafel Fentaw, Abebe Assefa, Gashaw Gebrewolde, Yonas Wuletaw, Ashenafi Assefa, Sisay Dugassa, Habte Tekie, Geremew Tasew

**Affiliations:** 1 Insect Science Stream, Department of Zoological Sciences, College of Natural and Computational Sciences, Addis Ababa University, Addis Ababa, Ethiopia; 2 Malaria and Neglected Tropical Diseases Research Team, Ethiopian Public Health Institute, P.O. Box: 1242, Addis Ababa, Ethiopia; 3 Clinical Bacteriology and Mycology Research Team, Ethiopian Public Health Institute, Addis Ababa, Ethiopia; 4 Vaccine and Diagnostic Research Team, Ethiopian Public Health Institute, Addis Ababa, Ethiopia; 5 Vector Biology and Control Research Unit, Aklilu Lemma Institute of Pathobiology, Addis Ababa, Ethiopia

**Keywords:** *Anopheles* species, Identification, Microflora, Midgut, Salivary glands

## Abstract

**Background:**

*Anopheles* mosquitoes are of great importance to human health. A number of studies have shown that midgut and salivary gland microflora have an impact on malaria parasite burden through colonization mechanisms, involving either direct *Plasmodium* microbiota interaction or bacterial-mediated induction of mosquito immune response. The objective of this study was to isolate and identify the microflora from the midgut and salivary glands of *Anopheles* species.

**Methods:**

A total of 20 pools (ten per pool) from insectary-reared and 56 pools (five per pool) of field-collected *Anopheles* mosquitoes were anesthetized by chloroform and dissected. 70% of ethanol was used for surface sterilization of mosquitoes and laboratory equipment, followed by rinsing *Anopheles* mosquitoes four times with 1X PBS. Each pool of dissected midgut and salivary gland sample was transferred in 1X PBS and squashed, incubated in the water bath and enriched in tryptic soya broth for 24 h at 35 ± 2 °C. As a control, the PBS solutions used to rinse the mosquitoes were also incubated in tryptic soya broth in the same conditions as the sample. After enrichment, a loopful of each sample was taken and inoculated on Blood, Chocolate, MacConkey, and Sabouraud Dextrose agar. Finally, the microbiota was isolated by colony characteristics, biochemical tests, and automated VITEK 2 Compact Analyzer.

**Results:**

From all field and laboratory mosquitoes, *Pseudomonas* was found to be the dominant microbiota identified from all species of *Anopheles* mosquitoes. *Acinetobacter* and *Klebsiellapneumonia* and other families of gram-positive and gram-negative bacteria were identified.

**Conclusions:**

A number of bacteria were isolated and identified. This is the first report on isolation and identification of microbiota from midgut and salivary glands of *Anopheles* species in Ethiopia. It can be used as a baseline for studying the relationship between microbiota and mosquitoes, and for the development of a new malaria biological control.

## Background

Malaria is an important vector borne disease caused by protozoan parasites in the genus *Plasmodium*. The *Plasmodium* species that cause human malaria are *P. falciparum, P. ovale, P. malariae, P. vivax* and *P. knowlesi*. These parasites are transmitted by infective bites of *Anopheles* mosquito vectors [1].

Malaria parasite transmission depends on the ability of mosquito vectors to support development of the parasites in the midgut and through to the infective sporozoite stages in their salivary glands [2]. However, commensal bacteria in the midgut can suppress parasite development and reduce the ability of mosquitoes to transmit the parasites to a new host, either by having direct anti-plasmodial effects or by stimulating basal immune responses of the mosquito against parasite development [3, 4]. The bacterial microflora in the midgut of mosquitoes have different effects on parasite development, which is likely to differentially affect the vector competence of *Anopheles* mosquito species and the probability of disease transmission [3].

Studies conducted to isolate and identify bacterial species in field-collected *Anopheles* and *Aedes* mosquitoes using microbe culturing techniques reported the presence of a wide range of bacterial taxonomic groups in the midgut [5, 6]. Similarly, a wide range of bacterial microflora including *Pseudomonas cepacia*, *Entrobacter agglomerans* and *Flavobacterium* species were identified in the midgut of three laboratory-reared *Anopheles* mosquito species [7]. Furthermore, the gut microflora varied depending on the sugar and blood feeding status of mosquitoes with reduced susceptibility of these mosquitoes to parasite development [7]. Moreover, midgut microfloral diversity depends on the ecological niche and geographical locations of vector mosquitoes. Straif and his colleagues [8] identified *Enterobacter agglomerans* and *Escherichia coli* as the most frequently isolated bacteria from midgut of field collected *An. gambiae* and *An. funestus* mosquitoes in Kenya and Mali.

The midgut microflora of *Anopheles* mosquitoes influence mosquito physiology, and also significantly alter vector competence [9]. As parts of the digestive system, the salivary glands harbor fewer bacterial microflora than the midgut of the mosquitoes [10]. However, Sharma et al. [11] showed that the salivary glands harbor more diverse microbial communities than the midgut in *An. culicifacies*.

Along with ecological factors such as sugar feeding, blood meals drastically alter mosquito gut microbial composition; these blood-fed midguts are enriched with *Pseudomonas* species [12]. These microbes in the gut of blood-fed mosquitoes may provide the additional genetic capacity to cope with oxidative stress due to catalase, manganese superoxidase dismutase, superoxide dismutase (Fe), heme oxygenase, alkyl hydroperoxide reductase (AhpC), and glutathione peroxidase enzymes in blood fed mosquitoes; such kind of genetic tolerance and fitness in mosquitoes is conferred by bacterial microflora in the stressful gut environment induced by a blood meal [12].

Thus, the resident microbiota in malaria vectors can enhance or suppress the development of the parasites that are to be transmitted to the mammalian host. Isolation and identification of microflora in the midgut and salivary glands of *Anopheles* mosquito species will provide data for further analysis of the tritrophic interaction of microbiota with the development of *Plasmodium* parasite in the mosquito vector for integrated malaria control in endemic areas. Therefore, the aim of this study was to assess, isolate and identify bacterial microflora from the midgut and salivary glands of laboratory-reared and field collected *Anopheles* mosquitoes in some malaria endemic areas of Ethiopia.

## Methods

### Insectary and laboratory procedures for *Anopheles arabiensis*

Mosquitoes were reared and maintained at 28 ± 2 °C/ and 80% relative humidity in Aklilu Lemma Institute of Pathobiology and Tropical and Infectious Disease Research Center (TIDRC) insectaries in Jimma. The insectaries were fitted with a simulated dawn and dusk machine which is essential for proper mating and feeding. For this study, 200 female *An. arabiensis* mosquitoes emerged after 24 h. were transferred and kept in paper cups. Sterile cotton swabs were soaked with 10% sterile sucrose solution and changed every hour and placed on the mosquito cages until dissection.

### Mosquito processing from field collected *Anopheles* mosquitoes

#### Study area

**Fig. 1:**
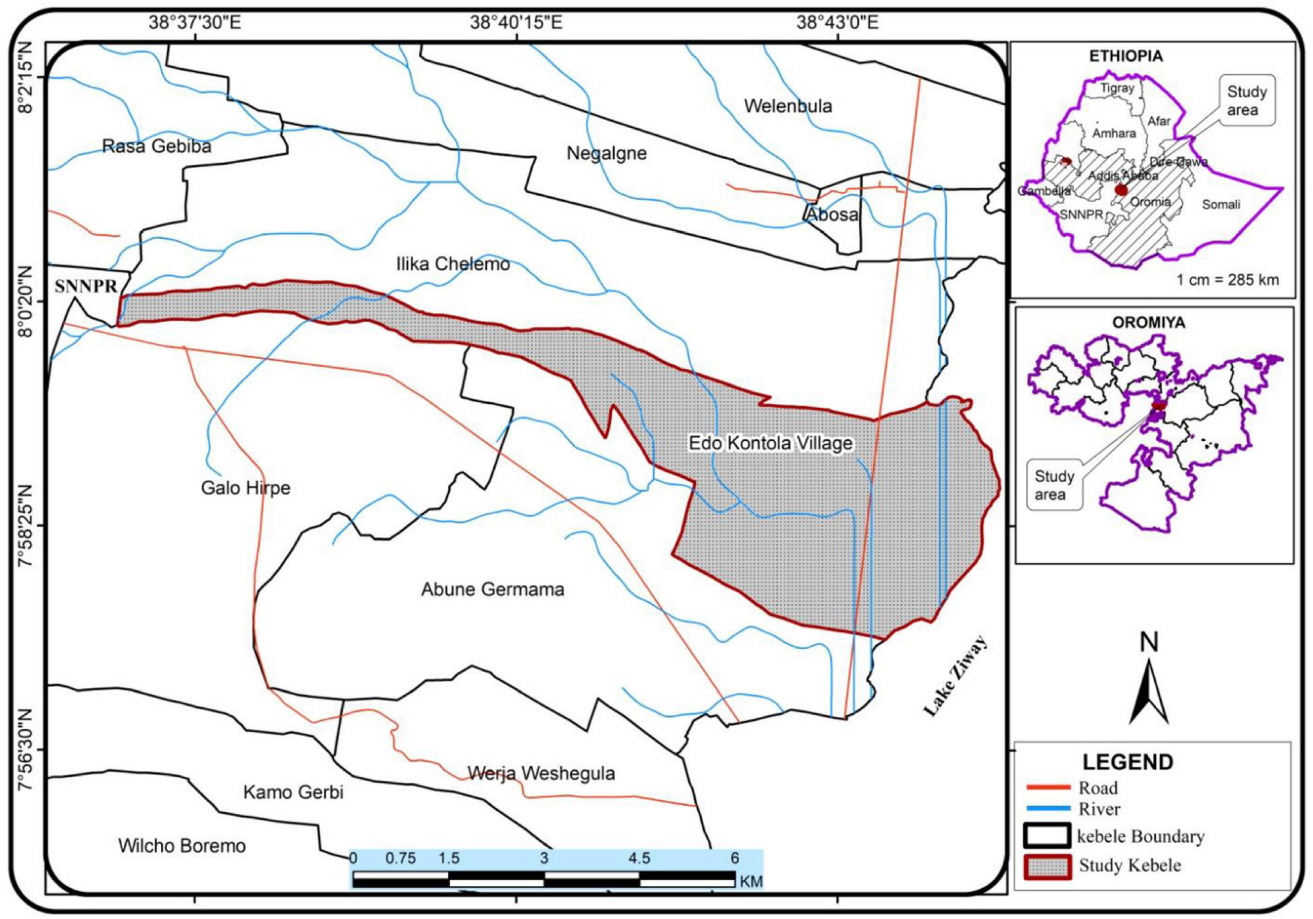
The study map Edo Kontola, Adami Tulu Jiddo KombolchaDistrict, Oromia, Ethiopia (Prepared by Bamlaku Amente, Department of Geography and Environmental Studies, Addis Ababa University, based on Census data from Central Statistics Agency, 2017, Addis Ababa Ethiopia)

This study was done in Edo Kontola village, Adami Tullu Jiddo Kombolcha District, South- Central Ethiopia (Fig. 1). Lake Zeway, is the main environmental feature of the area, covers an area about 434 km2 with an average depth of 4 m [13]. The sole economic activities of the society such as farms and fishing, are supported by this lake. During rainy season (June to October), people are usually cultivating maize and other cereal crops while onions, tomatoes, potatoes and green pepper are cultivated by irrigation during the dry season (November to May) as well as the wet season. ‘Mana Chita’ is the traditional African grass-thatched houses in which many of the inhabitants of the village live and some live in houses with corrugated iron roofs. The shoreline is the suitable potential mosquito breeding site in the area created and maintained by the lake [14]. Consequently, mosquito number increases after the rainy season and declines as the lake volume lessens during the dry months.

#### Ethical clearance

Ethical clearance was obtained from the College of Natural and Computational Science Institutional Review Board (CNS-IRB; IRB/031/2018) and Ethiopian Public Health Institute Institutional Review Board (EPHI-IRB; SERO-049-03-2017) to conduct this research.

#### Mosquito collection

Four houses were selected for the night caught *Anopheles* mosquitoes collection using CDC light traps and Human landing Catch (HLC) from indoor and outdoor respectively. CDC light traps were selected for four houses which close to the lakeshore and irrigation fields and mosquitoes were collected from the lakeshore within walking distance less than or equal to 1 km [15]. On the other hand, human volunteers were used for HLC mosquitoes collection which land on their exposed body parts from 19:00 to 06:00 for 50 min per each hour with a 10 min rest period.

For HLC, there were two collection shifts. One team collected from 19:00 to 24:00 whereas the second team from 24:00 to 06:00. To reduce position bias, two volunteers were rotated between indoor and outdoor positions and carried out the work in every hour. From each study house, there were 10 m gap between the position of outdoor collectors. Mosquitoes were captured as soon as they landed on the exposed foot to knee of each volunteer who did the collection using a flash light and mouth aspirator. Throughout the collection activities, the principal investigator coordinated and involved in the collection procedure and watched volunteers to ensure they did not fall asleep or get bitten by mosquitoes over the study nights [14]. In the following morning, mosquitoes were transported and their species was identified morphological characteristics using the standard identification key [16] at EPHI laboratory.

### Mosquito processing for laboratory reared and field collected *Anopheles* mosquitoes

A total of twenty pools of insectary-reared mosquitoes (10 per pool) and fifty-six pools of field- collected mosquitoes (5 per pool) were anesthetized by using a sterile cotton swab impregnated with chloroform. Throughout the dissection procedure in the laminar flow, the dissecting stereomicroscope (1X) working area, dissecting needles and forceps were dipped and sprayed in every dissection using 70% ethanol. Prior to the midgut and salivary gland dissection, each pool of mosquitoes was surface sterilized by washing in 70% ethanol followed by rinsing of each pool four times by 1X PBS. Each pool of dissected midgut and salivary glands was squashed and incubated in the water bath and transferred in 3 ml of 1X PBS along with control solutions and incubated in a water bath (27–31 °C) for 4 h until cultured in enriched Tryptic Soya Broth [17]. 1 ml of each pool and control solutions were transferred into 3 ml of Tryptic Soya Broth and incubated for 24 h. at 35 ± 2 °C.

A loopful of each pool of turbid broth was inoculated on Blood, Chocolate, Mac Conkey and Sabouraud dextrose agar and incubated in carbon dioxide incubator (Blood and Chocolate media) and aerobic incubator (Mac Conkey and Sabouraud dextrose media) for 24 h. at 35 ± 2 °C. For isolation of fungi from Sabouraud Dextrose media, the culture was incubated for fifteen days as fungi need more time to grow, and isolated based on their colony characteristics. Then, a wet film was used to identify yeast and other microbiota of fungi by observing under the microscope. Biochemical tests were done for other isolates from Blood, Mac Conkey, and Chocolate media.

Bacteria that could not be identified conventionally were analyzed and identified in VITEK 2 Compact (Biomerieux, France) which provides an automatic pipetting and dilution for identification of microbiota from the midgut and salivary glands of *Anopheles* mosquito samples. It has reagent cards with 64 wells for individual biochemical tests and has product type, lot number, expiration date, and a unique identifier that are linked to the sample. This machine requires pure colonies suspended in 3 ml of sterile aqueous 0.45% saline in 12x75mm clear plastic tubes. Turbidity was adjusted to 0.5 McFarland to get a standardized microbial load in the given sample. The tubes containing sample suspension were placed in a special rack (Cassette) which accommodates up to 10 tests. All cards were incubated at 35 ± 1 °C. Data was collected at 15-min intervals during the entire incubation period. In this machine, test reaction results appeared as “+” or “-” and an identification level above 95% considered as an excellent identification, and at least three organisms having the same biochemical reactions in the database or weak reaction and do not correspond to any taxon in the database considered as unidentified organisms.

## Results

### Microbiota composition in *Anopheles arabiensis* from insectary

Of 200 female *An. arabiensis*, a total of 110 microflora colonies from both midgut and salivary glands were counted. Of these, 97 represent 14 species of microflora including: *Klebsiella pneumonia*, *Saccharomyces cerevisiae*, *Serratia marcescens*, *Staphylococcus epidermidis*, *Pseudomonas luteola*, *Pseudomonas aeruginosa*, *Kocuria rhizophila*, *Streptococcus thoraltensis*, *Methylobacterium lacunta*, *Enterococcus casseliflavus*, *Kytococcus sendntarius*, *Lactococcus garvieae*, *Kocuria kristinae* and *Alloiococcus otitis*. The remaining 13 microflora colonies were identified at the genus level including *Pseudomonas* (nine isolates), *Bacillus* (one) and *Acinetobacter* (three). Table 1 shows the number of isolates and prevalence of microbiota identified from both midgut and salivary glands of *An. arabiensis* from the two insectaries. From a total of 47 identified microflora, 19% were *Saccharomyces cerevisiae* (Table 1). Similarly, *Pseudomonas* spp. comprised 19% of the total identified microflora. *Serratia marcescens* was the second most abundant microbiota comprised 13% of the total species which were isolated from 14 isolates. *Acinetobacter* and *Streptococcus thoraltensis* were comprised 6% of the total identified bacteria. *Lactococcus* and *Enterococcus* were identified in both midgut and salivary glands of *An. arabiensis* and comprise 11 and 4% respectively. *K. pneumonia*, *S. epidermidis*, *P. aeruginosa*, *Bacillus* species were the least identified microbiota. Besides, the results from the four control solutions were found to be *Erythrobacter* and *Bacillus* species analyzed by VITEK 2 Compact automation machine.

**Table 1:** The total number of isolates and microbiota identified from midgut and salivary glands of laboratory reared *An. arabiensis*

Bacterial species identified	Total # of isolates	Source of microbiota identified			Percent (*n* = 47)
		*Midgut*	*Salivary glands*	Total	
*Klebsiella pneumonia* ^††^	2	0	1	1	2
*Saccharomyces cerevisiae*	22	3	6	9	19
*Serratia marcescens* ^††^	14	3	3	6	13
*Staphylococcus epidermidis* ^†^	2	0	1	1	2
*Pseudomonas luteola* ^††^	2	1	0	1	2
*Pseudomonas aeruginosa* ^††^	2	1	0	1	2
*Kocuria rhizophila* ^†^	2	0	1	1	2
*Streptococcus thoraltensis* ^†^	8	2	1	3	6
*Methylobacterium lacunta* ^††^	2	0	1	1	2
*Enterococcus casseliflavus* ^†^	5	2	0	2	4
*Kytococcus sedentarius* ^†^	2	1	0	1	2
*Lactococcus garvieae* ^†^	12	3	2	5	11
*Kocuria kristinae* ^†^	2	1	0	1	2
*Alloiococcus otitis* ^†^	2	1	0	1	2
*Pseudomonas* spp*.*^††^	22	4	5	9	19
*Bacillus* spp*.*^†^	2	0	1	1	2
*Acinetobacter* spp*.*^††^	7	0	3	3	6
				47	

Note: † is gram positive bacteria species/genera, †† is gram negative bacteria species/genera

### Microbiota composition from field collected *Anopheles* mosquitoes species

During the three nights of collection of CDC light trap and human landing catches, a total of 280 female anopheline mosquitoes were captured. *An. zeimanni* (140) was the dominant species followed by *An. gambiae s.l.* (60), *An. pharoensis* (45) and *An. funestus* (35). Overall, 180 mosquitoes were captured outdoors and 100 indoors. A total of fifty six pools of mosquitoes, five mosquitoes per pool: five fed, five gravid and five unfed from midgut and salivary glands of each species of *Anopheles* mosquitoes were analyzed.

A total of 280 *Anopheles* mosquito midgut and salivary gland samples were analyzed. The samples were dissected and screened on four culture media resulting in a total of 68 microflora isolates. Fifty-three isolates were members of three genera and 5 species of gram negative bacteria, and the remaining 15 isolates of 4 species belonged to gram positive bacteria. Both gram negative and gram positive bacteria were isolated from fed, gravid and unfed *Anopheles* mosquitoes. Table 2 shows the abdominal conditions, sources of samples and *Anopheles* mosquitos’ species with their conventional biochemical test results along with their identified bacterial species.

**Table 2:** Biochemical test results of conventional culture method after 24 h incubation

*Anopheles* species	Abdominal condition	Sources of samples	Biochemical tests							Additional	Identified bacteria
			LDC	CIT	TSIA	SIM		UREA	H_2_S		
						Motility	Indole				
*Anopheles funestus*	Fed Gravid Unfed	Midgut Salivary gland	-ve	+ve	-ve	-ve	-ve	-ve	-ve	Cat^+^, Oxi^+^	*Pseudomonas* spp*.*^††^
	Gravid	S. gland	-ve	+ve	+ve	+ve	-ve	+ve	+ve	Cat^+^, Oxi^−^	*Citrobacter* spp.^††^
*Anopheles pharoensis*	Fed	S. gland	-ve	+ve	-ve	-ve	-ve	+ve	-ve	Cat^+^, Oxi^−^	*Klebsiella ozane* ^††^
			-ve	+ve	-ve	+ve	-ve	+ve	-ve	Cat^+^, Oxi^−^	*Providencia rettgeri* ^††^
	Gravid Unfed Unfed	Midgut	-ve	+ve	-ve	-ve	-ve	-ve	-ve	Cat^+^, Oxi^+^	*Pseudomonas* spp.^††^
		S. gland	+ve	+ve	-ve	-ve	-ve	-ve	-ve	Cat^+^, Oxi^−^	*Acinetobacter* spp.^††^
*Anopheles gambiae s.l*	Fed	S. gland	-ve	+ve	+ve	+ve	-ve	+ve	-ve	Cat^+^, Oxi^−^	*Enterobacter cloacae* ^††^
	Fed	Midgut S. gland	-ve	+ve	-ve	-ve	-ve	-ve	-ve	Cat^+^, Oxi^+^	*Pseudomonas* spp.^††^
	Gravid	Midgut S. gland	-ve	+ve	-ve	-ve	-ve	-ve	-ve	Cat^+^, Oxi^+^	*Pseudomonas* spp.^††^
		S. gland	+ve	+ve	+ve	-ve	-ve	+ve	-ve	Cat^+^, Oxi^−^	*Klebsiella pneumonia* ^††^
	Unfed	Midgut	-ve	+ve	-ve	-ve	-ve	-ve	-ve	Cat^+^, Oxi^+^	*Pseudomonas* spp.^††^
		S. gland	+ve	+ve	+ve	-ve	-ve	+ve	-ve	Cat^+^, Oxi^−^	*Klebsiella pneumonia* ^††^
*Anopheles zeimanni*	Fed	Midgut	+ve	+ve	-ve	-ve	-ve	-ve	-ve	Cat^+^, Oxi^−^	*Acinetobacter* spp.^††^
	Fed unfed gravid	Midgut S. gland	-ve	+ve	-ve	-ve	-ve	-ve	-ve	Cat^+^, Oxi^+^	*Pseudomonas* spp.^††^
	Gravid Unfed	S. gland Midgut	+ve	+ve	+ve	-ve	-ve	+ve	-ve	Cat^+^, Oxi^−^	*Klebsiella pneumonia* ^††^
	Gravid	S. gland	-ve	+ve	+ve	+ve	-ve	+ve	+ve	Cat^+^, Oxi^−^	*Citrobacter* spp.^††^
			+ve	+ve	-ve	-ve	-ve	-ve	-ve	Cat^+^, Oxi^−^	*Acinetobacter* spp.^††^
	Unfed	Midgut	-ve	+ve	+ve	+ve	-ve	+ve	+ve	Cat^+^, Oxi^−^	*Citrobacter* spp*.*^††^

Note: *LDC* Lysine decarboxylase, *CIT* Citrate, *TSIA* Triple Sugar Iron Agar, *SIM* Sulfur Indole Motility Media, *H*_*2*_*S* Hydrogen sulfide

†† is gram negative bacteria species/genera

A total of 9 bacterial species were identified from the four collected *Anopheles*species (Table 2 and Table 3). According to the number of isolated and identified species, it was shown that, *An.gambiae s.l.* had a higher diversity of bacteria with 7 species isolated from fed salivary glands, gravid salivary glands, fed and unfed midgut; divided in 6 genera of bacteria. In contrast, *An.funestus* had only two species from two genera identified: *Serratia* and *Streptococcus*. Surprisingly, *Serratia fonticola* was identified from unfed *An. pharoensis*, *An. zeimanni* and *An.gambiae s.l.*(Table 3). The genus *Pseudomonas*was the most frequently isolated bacteria in this study, found in 32 of the 68 isolates. In the conventional biochemical test, there were no fungal or yeast isolates found from Sabouraud Dextrose Agar media. From the control cultured rinsed solutions, *Erythrobacter* and *Bacillus* species were screened by VITEK 2 Compact automation machine. Moreover, conventionally unidentified microorganisms were identified by VITEK 2 Compact automation machine (Table 3).

**Table 3:** The summarized result of microbiota identified by Vitek 2 Compact from Adami Tullu, Jido Kombolcha District

*Anopheles* species	Feeding stages	Source of samples	Identified microbiota
*An. funestus*	Fed	sg	Unidentified
	Gravid	mg	*Ser. marcescens* ^††^
	Unfed	sg	*Str. thoraltensis* ^†^
*An. pharoensis*	Fed	mg	Unidentified
	Unfed	mg	*Staph. epidermidis* ^†^
	Unfed	sg	*Serratia fonticola* ^††^
*An. zeimanni*	Fed	mg	*Ser. marcescens* ^††^
	Gravid	mg	*Str. thoraltensis* ^†^
	Gravid	mg	*Str. thoraltensis* ^†^
	Fed	sg	*Str. thoraltensis* ^†^
	Unfed	sg	*Str. thoraltensis* ^†^
	Unfed	mg	*Ser. fonticola* ^††^
*An. gambiae s.l.*	Fed	mg	*Str. thoraltensis* ^†^
	Gravid	mg	*Str. mitis/oralis* ^†^
	Gravid	sg	*Staph. aureus* ^†^
	Unfed	mg	*Ser. fonticola* ^††^
	Unfed	sg	*Str. thoraltensis* ^†^
	Fed	mg	*Enterococcus faecium* ^†^
Control	The four PBS rinsed solutions		*Erythrobacter* and *Bacillus* species

Note: † is gram positive bacteria species, †† is gram negative bacteria species

mg is midgut, sg is salivary glands

## Discussion

The present study isolated and identified resident aerobic and salivary gland bacterial microbiota from laboratory reared *Anopheles arabiensis*, and from field collected of *Anopheles* mosquitoes, including *An. gambiae s.l.*, *An. zeimanni*, *An. pharoensis and An. funestus* from some endemic areas in Ethiopia.

The composition of isolates in this study showed that gram negative bacteria dominate the midgut and salivary gland flora of both laboratory reared and field collected *Anopheles* mosquito species (Table 1and 2). This agrees with earlier results from culture-based studies from different geographical areas on *An. stephensi*, *An. maculipennis* and other *Anopheles* mosquito species [18, 19], and on *Anopheles stephensi* and *Anopheles gambiae* [20]. Similar findings have also been reported from sequence-based studies on *An. darlingi* [21] and *An. gambiae* [15]. Some bacteria are common residents in the midugut and salivary glands of several *Anopheles* species from different ecological settings. For instance, the genus *Serratia* has been isolated from *An. stephensi* in India [22], from *An. culicifacies* in Iran [23], and from *An. gambiae* in Zambia [24]. This genus is identified from midgut and salivary glands of laboratory reared *An. arabiensis*, as well as from midgut of field collected gravid *An. funestus* and blood fed *An. zeimanni*, respectively, in the present study.

In the present study, *Pseudomonas* were the most frequently isolated bacteria from laboratory reared *An. arabiensis* (Table 1), and from field collected *An. funestus, An. pharoensis, An. zeimanni* and *An. gambiae s.l*. (Table 2). This genus has been commonly isolated in several mosquito vectors in Asia and the Americas [23, 25]. However, it was isolated at a low level in mosquitoes in Kenya [3]. Previous findings reported the genera *Pseudomonas*, *Serratia* and *Acinetobacter* in the midgut while *Pseudomonas* and *Acinetobacter* were identifiedfrom the salivary glands of *Anopheles* mosquitoes [26].

In addition to bacteria, fungi are common microbiota frequently isolated from the mosquito larvae in aquatic breeding habitats and, from adults either through ingesting fungi in a sugar meal or through external physical contact with fungal spores [27]. As a result, the mosquito midgut and other tissues have been parasitized by common fungal genera *Beauveria and Metarhizium* [28]. In this study, a total of nine *Saccharomyces* yeasts species were identified from 22 microflora colonies of laboratory reared *An. arabiensis* (Table 1). Of these, six *Saccharomyces* species were isolated from salivary glands and three *Saccharomyces* species were isolated from the midgut cultures of *An*. *arabiensis*. These results are in line with the findings on isolation of yeasts from laboratory reared and field collected *Anopheles* mosquitoes [29].

In this study, *Serratia marcescens* and *Streptococcus mitis* were identified from midgut cultures of gravid *An. funestus* and *An. gambiae s.l.,* respectively. In addition, *Staphylococcus epidermidis* and *Enterobacter cloacae* isolates were identified from the midgut of unfed *An. pharoensis*, salivary glands of laboratory reared *Anopheles arabiensis,* and from midgut of field collected gravid *An. gambiae s.l*. In contrast to these findings, *Staphylococcus epidermidis* and *Enterobacter cloacae* were the dominantisolated bacteria from the midgut of fed *An. gambiae* and based on molecular analysis and cultures of fed and gravid *An. gambiae* microbiota indicated that *Serratia marcescens* and *Streptococcus mitis* were the dominant isolates from *An. gambiae* [30].

*Anopheles* mosquitoes harbor diverse microbiota which affects their physiology, metabolism and immune processes. Midgut and salivary gland microbiota in mosquitoes also affect the outcome of the mosquito infection with the *Plasmodium* parasites [31]. A recent study based on metagenomic analysis combined with culture methods provided an insight in to the role of the *Anopheles*-associated microbiota in modulating the immune system of the vector mosquitoes and affect parasite development and disease transmission. Among these *Anopheles*-associated microbiota: *Lactococcus garvieae*, *Kocuria kristinae*, *Enterococcus casseliflavus* and *Methylobacterium lacunta* were identified from the body of *Anopheles* mosquitoes from different geographical locations [32]. These bacterial species were consistently isolated and identified from salivary gland and midgut cultures of laboratory reared *An. arabiensis* in the present study.

## Conclusions

This study describes isolation and identification of microbiota in the midgut and salivary glands of *An. arabiensis* from insectary and field collected *Anopheles* species. To our knowledge, it is the first study providing an in depth description of the microbiota diversity in midgut and salivary glands of *Anopheles* mosquitoes. Our findings indicated that, *Pseudomonas* species had the dominant microbiota identified from all species of field collected *Anopheles* mosquitoes and insectaries. Moreover, *An. arabiensis* has a more diversified microbiota compared to the other species.

## Abbreviations

CDC: Center for Disease Control and Prevention
CIT: Citrate
EPHI: Ethiopian Public Health Institute
HLC: Human Landing Catch
LDC: Lysine decarboxylase
PBS: Phosphate Buffer Saline
SIM: Sulfur indole motility media
TIDRC: Tropical and Infectious Disease Research Center
TSIA: Triple sugar iron agar
